# Engineering Fc heterodimerization via asymmetric β-strand reconfiguration for bispecific antibody assembly

**DOI:** 10.1186/s13036-026-00717-x

**Published:** 2026-06-18

**Authors:** Arkaitz Cano, Izaskun Morillo, Ainhoa Goenaga, María Elena Laugieri, Ander de Blas, Eunate Valdaliso-Díez, June Ereño-Orbea, Beatriz Apellaniz, Edurne Rujas

**Affiliations:** 1 Department of Physiology, Faculty of Pharmacy, University of the Basque Country (UPV/EHU), Vitoria-Gasteiz, 01006 Spain; 2 Instituto Biofisika (UPV/EHU, CSIC), University of the Basque Country, Leioa, 48940 Spain; 3 Department of Pharmacy and Food Sciences, Faculty of Pharmacy, University of the Basque Country (UPV/EHU), Vitoria-Gasteiz, 01006 Spain; 4 Cancer Glycoimmunology Lab, Center for Cooperative Research in Biosciences (CIC bioGUNE), Basque Research and Technology Alliance (BRTA), Derio, 48160 Spain; 5 Ikerbasque, Basque Foundation for Science, Bilbao, 48013 Spain; 6 Bioaraba Health Research Institute, Microbiology, Infectious Disease, Antimicrobial Agents, and Gene Therapy Group, Vitoria-Gasteiz, 01009 Spain

**Keywords:** Bispecific antibody, Protein engineering, Heavy chain pairing, Heterodimerization

## Abstract

Bispecific antibodies (bsAbs) have unlocked new therapeutic modalities by enabling dual antigen targeting, offering transformative potential in oncology and autoimmune diseases. However, the efficient production of full-length IgG-like bsAbs remains hindered by mispairing of heavy and light chains, yielding heterogeneous, low-purity products. Here, we present an Fc engineering strategy, termed StrandLock, that enables the controlled assembly of bsAbs through asymmetric β-strand redistribution at the CH3 dimer interface, in which the β-strand segment (residues N390–S400) is duplicated in one heavy chain and deleted from the partner. This structural asymmetry, combined with targeted point mutations, promotes obligate heterodimerization while suppressing homodimer formation. Importantly, these modifications enable the correct assembly of bsAbs with purities approaching 99% and allow streamlined manufacturing via a single-step affinity purification process comparable to that used for monoclonal antibodies. The engineered bsAbs retain key Fc effector functions and exhibit comparable in vivo pharmacokinetics to native IgG. Structural analysis confirms preservation of the CH3 fold and native-like interfacial packing. Despite a modest reduction in thermal stability, the molecules remain aggregation-resistant and functionally robust under accelerated stress conditions. We further demonstrate the versatility of the StrandLock design across multiple antibody pairs underscoring its utility as a strategy for bsAb production.

**Supplementary Information:**

The online version contains supplementary material available at 10.1186/s13036-026-00717-x.

## Introduction

Over the past three decades, monoclonal antibodies (mAbs) have revolutionized the treatment of a wide range of diseases, establishing themselves as a cornerstone of modern therapeutics. Their clinical success has been driven not only by their high specificity and favorable safety profiles, but also by continuous improvements through innovations in protein engineering. Among these, bispecific antibodies (bsAbs) have emerged as a particularly promising class of molecules, capable of simultaneously recognizing two distinct antigens or epitopes. This dual-targeting capacity unlocks therapeutic mechanisms that go beyond those of conventional mAbs, including the redirection of immune effector cells to tumor sites, the simultaneous blockade of signalling pathways, and enhanced selectivity for diseased tissues [1]. These properties have expanded the therapeutic landscape of antibody-based interventions and have been validated through an increasing number of preclinical and clinical studies [2].

A key factor underlying the functional diversity of bsAbs is the range of molecular formats available for their design [3]. These formats vary in multiple structural parameters, such as size, symmetry and valency, each of which can significantly influence their pharmacological behavior. One of the most relevant differences in architecture among bsAbs is whether they include the crystallizable fragment (Fc) domain or not. Based on this distinction, bsAbs are broadly classified into two categories: Fc-containing (IgG-like) and Fc-free (non-IgG-like) formats.

The Fc fragment not only enhances antibody stability but also plays a pivotal role in its pharmacokinetic profile through neonatal Fc receptor (FcRn)-recycling [4]. Additionally, the Fc domain mediates immune-modulatory functions by engaging Fc gamma receptors (FcγRs) and activating complement pathways [5]. These interactions enable key immune effector mechanisms, including antibody-dependent cellular cytotoxicity (ADCC), antibody-dependent cellular phagocytosis (ADCP), and complement-dependent cytotoxicity (CDC). Collectively, these attributes render IgG-like bsAbs particularly well suited for therapeutic indications requiring prolonged systemic exposure and immune system engagement, such as oncology and chronic inflammatory diseases.

In contrast, non-IgG-like bsAbs, such as bispecific T-cell engagers (BiTEs), diabodies and dual-affinity retargeting proteins (DARTs) [6], consist of antibody-derived fragments joined by flexible linkers. While these compact formats are advantageous in terms of modular design, tissue penetration and ease of engineering, they generally suffer from limited in vivo stability, short half-life and increased aggregation risk [7, 8]. For example, blinatumomab, an FDA-approved BiTE targeting CD19 and CD3 for B-cell acute lymphoblastic leukemia [9], requires continuous intravenous infusion due to its short serum half-life [10], posing challenges in clinical administration. Pharmacokinetics, solubility and stability of these bispecific molecules can be improved with the addition of the Fc fragment [11–13].

Despite their pharmacological advantages, the development of full-length IgG-like bsAbs poses major manufacturing challenges due to the intrinsic symmetry of IgG molecules. In native antibodies, Fc dimerization is mediated by symmetric interactions between the CH3 domains, i.e., the C-terminal constant domains of the IgG heavy chains (HCs), which inherently favor homodimer formation. When two distinct HCs and two distinct light chains (LCs) are co-expressed, stochastic pairing occurs at both the CH3-mediated Fc interface and the Fab constant domain governing HC-LC association. As a consequence, random assembly generates up to nine possible molecular species, of which only a small fraction (~ 11%) represents the correctly assembled bispecific heterodimer [14]. The remaining off-target species, including monospecific homodimers and mispaired antibodies, are difficult to separate because of their closely related physicochemical properties, ultimately reducing both the yield and purity of the final product.

To mitigate HC mispairing, a variety of Fc engineering strategies promote selective HC heterodimerization by introducing asymmetry at the CH3-CH3 interface. The “Knobs-into-Holes” (KiH) approach relies on complementary steric mutations in the CH3 domains, whereby bulky amino acid substitutions on one heavy chain (“knobs”) are paired with compensatory cavities (“holes”) [15]. Electrostatic steering strategies, in contrast, introduce oppositely charged residues to bias correct chain pairing [16]. Beyond point mutations, additional approaches exploit larger sequence or structural asymmetries at the CH3 interface. These include cross-isotype or domain-exchange strategies, such as Strand-Exchange Engineered Domains (SEED), in which β-strands from different immunoglobulin isotypes are combined within each HC to create complementary heterodimeric interfaces [17], and BEAT (Bispecific Engagement by Antibodies based on the T-cell receptor) designs, which reengineer the CH3 domains to mimic the asymmetric constant domain interfaces of the TCR α/β heterodimer, thereby promoting preferential HC heterodimerization [18]. Alternative strategies take advantage of the intrinsic Fab-arm exchange properties of IgG4 antibodies, using controlled redox conditions and hinge engineering to drive the formation of bispecific molecules [19]. While these approaches have significantly improved bsAb assembly and yields, homodimer contamination and incomplete control over chain pairing remain persistent challenges [16, 20, 21] limiting manufacturing efficiency and scalability.

In this study, we describe an Fc heterodimerization strategy that promotes selective pairing of distinct HCs through asymmetric β-strand redistribution at the CH3 domain interface (residues N390–S400). This approach, termed StrandLock, introduces structural asymmetry and interdependent folding between the HCs, thereby favoring heterodimer assembly. To further suppress undesired homodimer formation, targeted point mutations were introduced within the newly formed interfaces, resulting in near-complete heterodimerization. Our data demonstrate that the engineered Fc retains key biophysical and functional properties and supports the efficient production of high-purity bsAbs across a range of antibody combinations. Collectively, these findings support StrandLock as a structurally distinct approach for Fc heterodimerization that complement existing bsAb engineering strategies.

## Results

### Asymmetric Fc β-strand redistribution enables a distinct strategy for bsAb assembly

**Fig. 1:**
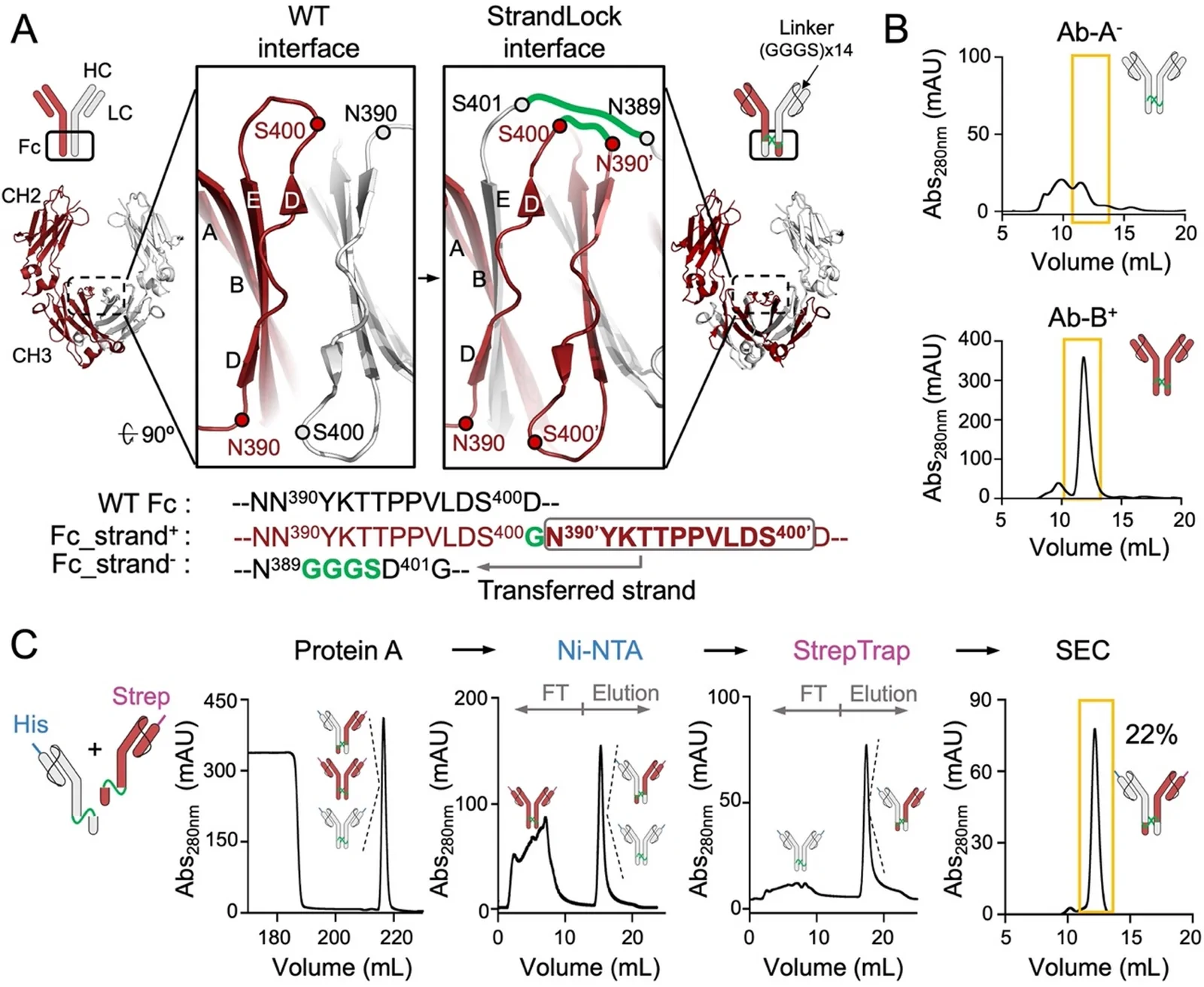
Design and validation of the StrandLock technology for bsAb assembly. (**A**) Schematic representation of the β-strand redistribution approach. Left: Fc region of a WT IgG (PDB ID 3AVE), composed of two identical HCs (shown in gray and dark red), illustrating its natural symmetry. Right: Engineered Fc domain designed to break this symmetry and to promote heterodimer formation. *Insets*: Close-up views of the arrangement of the two β-strands D (residues N390-S400 denoted by the prime (′)) at the Fc interface. In the StrandLock design, both β-strands D are connected via a glycine residue (green), generating the modified chain denoted Fc_strand^+^ (dark red). The open ends created by this modification (residues 389 and 401) are joined using a GGGS linker (green), yielding Fc_strand^−^ (gray). To prevent LC-HC mispairing, scIgG constructs with flexible linkers were used. The resulting antibodies were designated Ab-B⁺ (dark red), reflecting duplication of β-strand D, and Ab-A⁻ (gray), reflecting its absence. (**B**) SEC profiles of individually expressed, engineered monospecific antibodies after affinity purification. Yellow boxes indicate the elution volumes corresponding to properly assembled dimeric IgG species. Data show one representative experiment of three replicates. (**C**) Sequential purification of the bsAb using orthogonal affinity tags: a His-tag (blue) on Ab-A^−^ and a Strep-tag (pink) on Ab-B^+^. FT: flow-through

To promote selective HC pairing, we designed an Fc strategy based on asymmetric β-strand redistribution, aimed at disrupting the intrinsic two-fold symmetry of the Fc homodimer **(**Fig. 1A**)**. This strategy targets the CH3-CH3 dimerization interface which is stabilized by a combination of hydrophobic packing, symmetric electrostatic interactions and inter-chain hydrogen bonds [22, 23]. Within this interface, a region spanning residues N390–S400, centered on β-strand D, was identified as a strategic target for modification. Owing to the antiparallel arrangement of the two β-strands, the C-terminal residues of this region in one Fc chain lies approximately 7 Å from the N-terminal residues of the corresponding region in the opposing chain, providing a geometrically favorable site for interchain connection via short, flexible linkers **(**Fig. 1A and Fig. S1A). By asymmetrically redistributing β-strand D from one chain to the other, this strategy redirects the folding trajectory, allowing a segment from one Fc chain to contribute directly to the folding of its partner chain (Fig. S1B). This rearrangement is expected to break the intrinsic two-fold symmetry of the homodimer and provide a structural basis for selective heterodimer formation.

To test this concept, β-strand D was asymmetrically redistributed between the two Fc chains. As a proof of concept, the strand was taken from the Fc of atezolizumab (anti-programmed death-ligand 1(PD-L1) IgG) to generate the Fc_strand^−^ variant, and inserted into the Fc of denintuzumab (anti-cluster of differentiation 19 (CD19) IgG) to produce the Fc_strand^+^ variant. To preserve local flexibility and promote proper folding, a GGGS linker was used to reconnect the ends of the (-) chain after strand removal, while a single glycine (G) was introduced to connect the inserted strand within the (+) construct (Fig. 1A). Both constructs were expressed as single-chain IgGs (scIgGs), with the LC fused to the N-terminus of the HC via a 70-amino-acid flexible linker [(GGGGS)₁₄], ensuring proper folding and preventing LC mispairing [24]. Full-length scIgG constructs incorporating Fc_strand^–^ and Fc_strand^+^ are hereafter designated Ab-A^–^ and Ab-B^+^, respectively.

When expressed individually, Ab-A^−^ exhibited low yields and minimal properly folded antibody as assessed by size-exclusion chromatography (SEC), indicating that deletion of β-strand D effectively prevents homodimer formation (Fig. 1B, top). In contrast, Ab-B^+^ yielded substantial amounts of correctly folded antibody, demonstrating insertion of the strand is structurally tolerated and does not strongly interfere with CH3-CH3 interactions (Fig. 1B, bottom).

Co-expression of both constructs was used to evaluate heterodimer formation. Orthogonal affinity tags, His-tag on Ab-A^−^ and Strep-tag on Ab-B^+^, enabled selective purification. Sequential affinity purification revealed significant protein loss in the His-tag flow-through, but not in the Strep-tag column, further supporting that Ab-A^−^ is unable to homodimerize, while Ab-B^+^ retains this capability (Fig. 1C). Importantly, ca. 20% of the total IgG product was the desired bispecific antibody, demonstrating compatibility of β-strand D transfer with heterodimer formation.

### Rational Fc engineering enables complete heterodimerization and suppression of homodimers

Although β-strand redistribution provides a viable framework for bsAb generation, additional molecular refinements are necessary to achieve stringent selective heterodimerization and complete suppression of homodimer formation. To this end, targeted mutations were introduced into the Fc regions of Ab-A^−^ and Ab-B^+^ to enforce obligate assembly between distinct HCs.

**Fig. 2:**
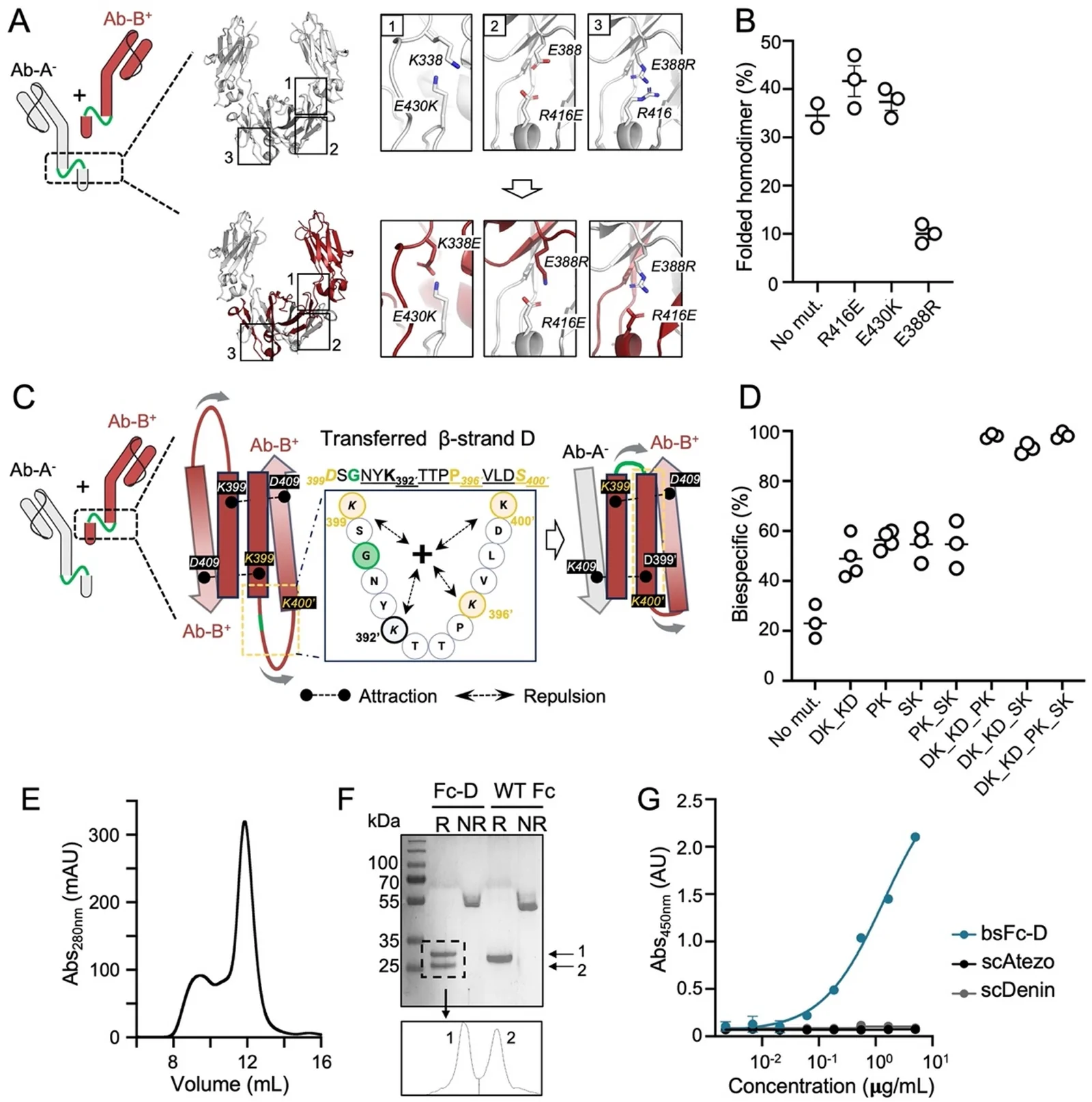
Optimization of asymmetric Fc chains to prevent homodimer formation. (**A**) CH3 interfaces in the engineered Fc. Three new inter-chain contact regions generated upon β-strand D transfer are highlighted. Residues proposed for mutation to induce electrostatic repulsion in the Ab-A⁻ homodimer (gray) or attraction between Ab-A⁻ and Ab-B⁺ (dark red) are shown. (**B**) Percentage of properly folded monospecific Ab-A^−^ mutants. (**C**) Schematic representation of how the additional β-strand can form a flexible loop outside the Fc core, enabling homodimer formation of Ab-B⁺ (dark red). The close-up view highlights residues of the transferred β-strand D (indicated by a prime, ′) that were targeted for lysine substitution (yellow) to generate electrostatic repulsion (double-headed arrow) with K392′ (black circle), thereby hindering homodimerization and promoting heterodimer assembly with Ab-A⁻ (gray). (**D**) Quantification of bsAb formation after co-transfection of Ab-A^−^ (I253A mutant to prevent protein A binding) with Ab-B⁺ variants carrying single or combined lysine substitutions. (**E**) SEC profile of the bsAb (bsFc-D) assembled from optimized variants: Ab-B^+^ carrying D399K, K409D, P396’K, and R416E; and Ab-A^−^ carrying E388R. (**F**) SDS-PAGE of Fc fragments from bsFc-D and WT IgG under reducing (R) and non-reducing (NR) conditions. Densitometry of the two R bands in bsFc-D is shown. (**G**) Simultaneous binding of bsFc-D to both target antigens compared with monospecific scAtezolizumab (scAtezo) and scDenintuzumab (scDenin). Representative data from > 3 replicates; mean ± SD of two technical replicates

For the Ab-A^−^ construct, the new interchain interfaces created by the transferred β-strand architecture provided an opportunity to modulate electrostatic interactions. Analysis of the 3D structure of the CH3 domain revealed three regions containing charged residues suitable for asymmetric engineering (Fig. 2A). Accordingly, three single-point mutations: E388R, R416E and E430K were individually introduced into Ab-A^−^. Each substitution was designed to introduce unfavorable electrostatic interactions with residues R416, E388 and K338, respectively, upon Ab-A^−^ homodimer formation, thereby destabilizing self-association. To enable productive heterodimerization in subsequent co-expression experiments, complementary charge substitutions (R416E, E388R and K338E, respectively) were introduced for the partner Ab-B^+^ construct **(**Fig. 2A**)**. Among the individually evaluated Ab-A^−^ variants, the E388R mutation was the most effective, resulting in over 90% aggregation, as determined by SEC peak integration, and negligible formation of correctly folded IgG, consistent with effective suppression of Ab-A^−^ homodimerization (Fig. 2B and Fig. S2A).

Regarding the Ab-B^+^ construct, it was hypothesized that the transferred β-strand D extends beyond the CH3 core and could form a flexible loop compatible with homodimer assembly (Fig. 2C). To selectively destabilize this potential conformation, lysine substitutions were introduced at positions P396′, S400′ (within the transferred β-strand D as denoted by the prime, ′), as well as at D399, with the aim of introducing electrostatic repulsions with K392′ (Fig. 2C). Notably, D399 participates in a stabilizing salt bridge with K409; therefore, mutation of D399 to lysine (D399K) was accompanied by reciprocal mutation of K409 to aspartate (K409D), generating a compensatory charge pair (DK_KD) to preserve heterodimer compatibility.

To assess whether these rationally designed mutations on Ab-B^+^ effectively promoted selective heterodimer formation, Ab-B^+^ variants were co-expressed with Ab-A^−^. To facilitate bsAb quantification and reduce purification complexity, an I253A mutation was introduced into Ab-A^−^ to disrupt protein A binding [25, 26]. Given the intrinsically low expression of Ab-A^−^ homodimers, this modification effectively removed them from the final product pool (Fig. S2B) allowing rapid and accurate assessment of bsAb recovery without additional affinity purification (Fig. S2C).

BsAb yields across the panel of Ab-B^+^ mutants were quantified using this streamlined purification strategy. Individually, the P396′K (PK), S400′K (SK), and DK_KD mutations each increased the proportion of bsAb from ca. 20% to 50–55%. This improvement corresponded to an increase in expression yields from 2.8 mg/L in the absence of mutations to ~ 3.3 mg/L for PK, and to higher yields of 5.9 and 5.2 mg/L for the SK and DK_KD variants, respectively. Combining SK and PK did not further improve either yield or the percentage of bsAb compared with the individual mutations. In contrast, combining DK_KD with PK, or with both SK and PK, increased bsAb production to 99% while sustaining high expression levels (~ 8 mg/L). By comparison, the DK_KD_SK combination reached 93% bsAb but at reduced yields (~ 5 mg/L), underscoring the need to consider both heterodimer enrichment and total production when evaluating variant performance (Fig. 2D and Fig S2D). Consistent with these results, expression of the individual Ab-B^+^ variants that drive near-complete bsAb formation produced no detectable protein A recovery, confirming effective suppression of Ab-B homodimerization (Fig. S2E). This contrasts with established heterodimerization platforms such as KiH [15], electrostatic steering [16], or Zymeworks [27], where expression of the individual monospecific chains can still lead to homodimer formation. (Fig. S3). These observations reinforce that such platforms are designed to bias assembly toward the desired heterodimer, rather than strictly enforcing opposite-chain pairing, and thus, homodimeric contaminants remain possible.

Based on these results, atezolizumab (Ab-A^−^) carrying the E388R mutation and denintuzumab (Ab-B^+^) containing the DK_KD, PK, and charge-complementary R416E mutations were co-transfected. This approach yielded a properly folded bsAb (hereafter referred to as bsFc-D) with expression levels approximately 8 mg/L (Fig. 2E). Papain digestion of purified bsFc-D, followed by Fc fragment isolation and SDS-PAGE under reducing conditions, revealed two distinct chains in the Fc fragment (hereafter referred to as Fc-D) differing by ~ 2 kDa (Fig. 2F). This size difference reflects either the duplication or the absence of β-strand D. In contrast, the Fc fragment from the wild-type antibody (WT Fc) showed a single band corresponding to identical chains. Densitometric analysis confirmed near-equimolar levels of both Fc chains in Fc-D (Fig. 2F), consistent with efficient bsAb assembly without monospecific contaminants. Non-reducing SDS-PAGE demonstrated intact disulfide bonds in both WT Fc and Fc-D, confirming preservation of structural integrity (Fig. 2F). Finally, sandwich ELISA verified dual-target binding of bsFc-D to PD-L1 and CD19, confirming functional bispecificity (Fig. 2G).

### Structural integrity of the CH3 domain is retained after asymmetric β-strand redistribution

Next, we determined the crystal structure of the engineered Fc-D fragment, generated by papain digestion of bsFc-D. Crystals of Fc-D diffracted to 2.064 Å resolution and belonged to the orthorhombic space group P2₁2₁2₁, with one Fc dimer per asymmetric unit (Table S1).

**Fig. 3:**
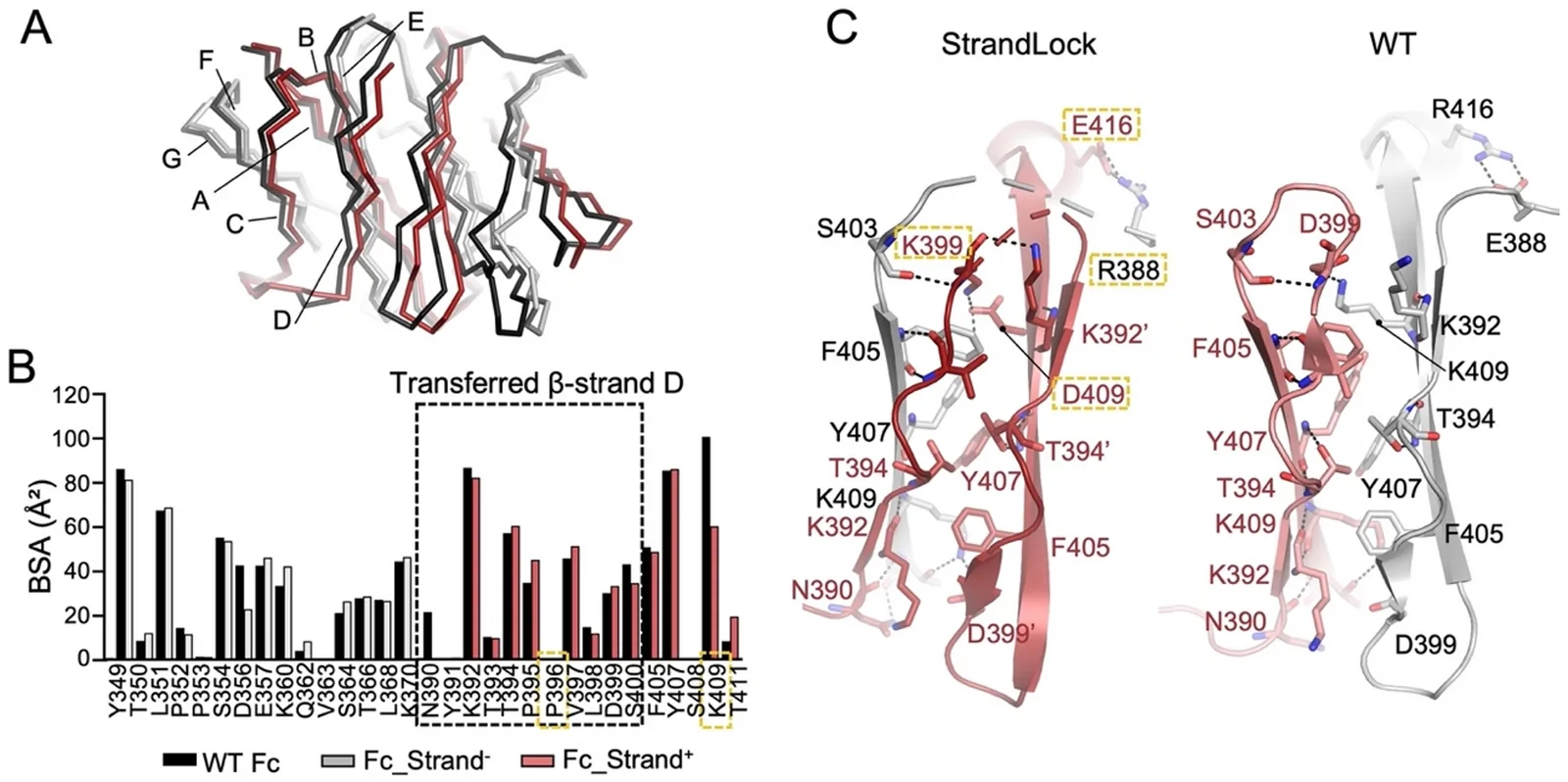
Crystal structure of the Fc-D domain. (**A**) Ribbon representation of the superposition of the CH3 domains from Fc-D (red and gray) and WT Fc (black). (**B**) BSA comparison of interface residues involved in Fc dimerization in the WT Fc and their corresponding residues in the engineered Fc-D structure. Residues from β-strand D are indicated by a black dashed box, while mutated residues are marked with yellow dashed boxes. (**C**) Close-up view of the β-strands D and E from each monomer within the CH3 domain of the Fc-D (left) and WT (right, PDB ID: 3AVE) crystal structure. Hydrogen bonds are indicated by dashed black lines, and mutated residues are highlighted with dashed boxes

The engineered Fc-D adopts a CH3 dimer architecture that closely resembles that of the WT Fc (Fig. S4A). Structural superposition of the engineered and WT CH3 (PDB ID: 3AVE) domains yielded a backbone r.m.s.d. of 0.359 Å, indicating preservation of the global CH3 fold and compatibility of the StrandLock strategy with the native Fc architecture (Fig. 3A and Fig. S4B). Consistently, interface analysis showed that the buried surface area (BSA) is comparable to that of the WT Fc, indicating that native packing interactions are maintained (Fig. 3B).

The transferred β-strand D is clearly resolved and properly incorporated into the CH3 dimer interface, where it adopts a canonical β-sheet conformation consistent with the native topology **(**Fig. 3C**)**. No electron density was observed for residues S400 and the G linker in Fc_Strand⁺ or N389 and the GGGS linker in Fc_strand⁻, consistent with the intrinsic flexibility of this region (Fig. S4C).

Comparison with the WT structure reveals localized rearrangements in specific intramolecular interactions arising from the engineered substitutions. Specifically, substitution of D399 with K introduces new intrachain hydrogen-bonding interactions, with K399 forming contacts with D421 and K404’ within the Fc_Strand⁺ chain **(**Fig. 3C**)**. Despite these localized rearrangements, the principal interactions stabilizing the CH3 dimer interface remain conserved. In particular, the hydrophobic π–π stacking interaction between opposing Y407 residues is preserved, as is the interaction involving F405 and K409 observed in the WT structure, indicating maintenance of the core interface architecture **(**Fig. 3C**)**.

### Fc-mediated activity and systemic exposure are maintained in bsFc-D

**Fig. 4:**
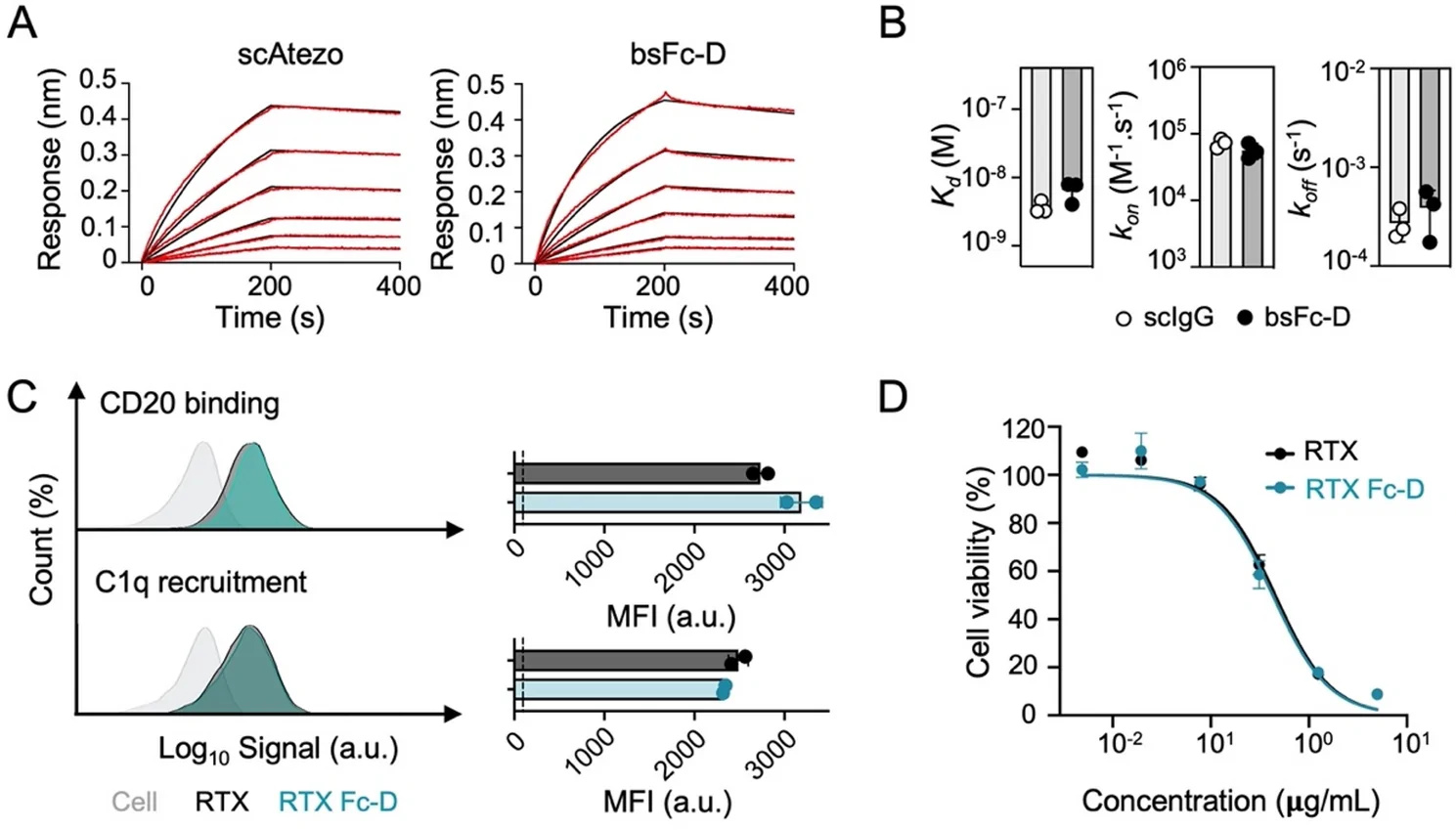
Fc-mediated function of bsFc-D compared with WT IgGs. (**A**) Binding kinetics of bsFc-D and scIgG atezolizumab (WT Fc) to human FcγRI. Red lines: raw data; black lines: global fits. (**B**) The dissociation constant (*K*_*d*_), association rate (*k*_*on*_), and dissociation rate (*k*_*off*_) are shown for comparison. Data are shown as mean ± SD from three independent replicates. (**C**) Representative flow cytometry plots of RTX variants carrying WT Fc (black) or engineered Fc (RTX Fc-D, cyan), showing binding to Daudi B cells (top left) and C1q recruitment (bottom left). Right: quantification of mean fluorescence intensity (MFI) from two independent replicates ± SD. Dotted line: MFI in the absence of antibody. (**D**) Cell viability of Daudi cells treated with RTX variants in the presence of human serum as a measure of CDC. Data represent mean ± SD from two biological replicates

To determine whether this strategy alters core functional or biophysical properties of the IgG, key effector functions were evaluated. As a direct measure of Fc-mediated activity, binding to FcγRI was first evaluated by biolayer interferometry (BLI). The engineered Fc displayed binding kinetics and nanomolar affinities indistinguishable from those of scAtezolizumab containing a WT Fc domain **(**Fig. 4A, B**)**, indicating preserved receptor engagement. In addition, we examined the capacity to elicit complement-dependent cytotoxicity (CDC). For this purpose, the engineered Fc was incorporated into rituximab (RTX), a type I anti-CD20 antibody that represents a benchmark for robust complement activation [28]. The StrandLock strategy effectively impaired the formation of homodimer species and enabled the generation of RTX Fc-D (Fig. S5A), which binds CD20 on Daudi cells with levels comparable to WT RTX, as assessed by flow cytometry (Fig. 4C, top panels). C1q recruitment, the initiating step of the classical complement pathway, was subsequently measured and found to be equivalent between RTX Fc-D and the WT variant (Fig. 4C, bottom panels). Building on these results, CDC assays in human serum demonstrated comparable dose-dependent lysis of target cells **(**Fig. 4D**)**, indicating that Fc effector functions were preserved following asymmetric β-strand redistribution.

**Fig. 5:**
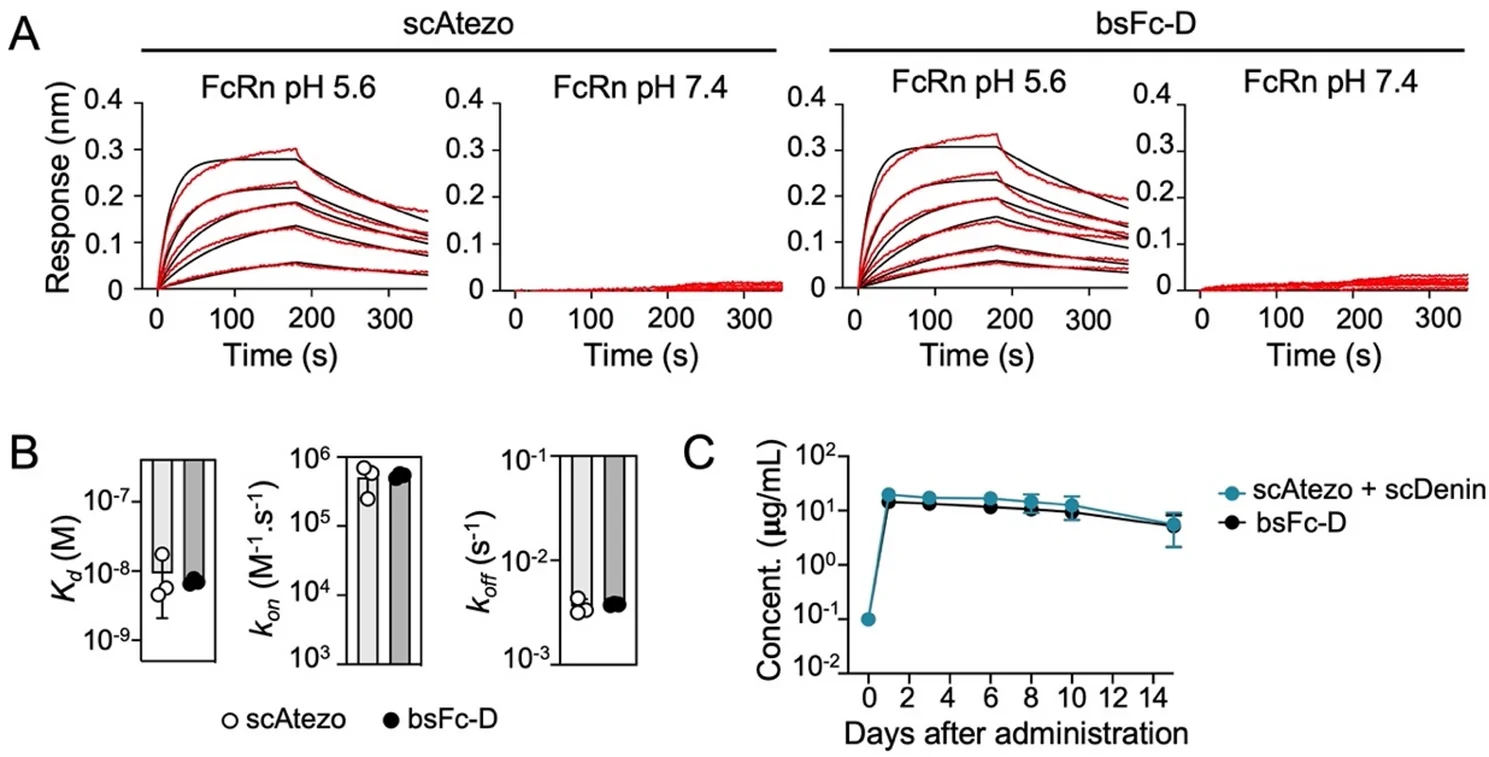
FcRn binding and in vivo exposure of bsFc-D. (**A**) Concentration-response curves for FcRn binding of bsFc-D and scIgG atezolizumab (WT Fc) at endosomal (pH 5.6) and physiological (pH 7.4) conditions. Red: raw sensograms; black: global fits. (**B**) Binding kinetics from panel A, including dissociation constant (*K*_*d*_), association rate (*k*_*on*_), and dissociation rate (*k*_*off*_). Data represent mean values from three biological replicates. (**C**) Serum antibody levels following intraperitoneal injection (5 mg/kg) in SCID mice. Mean ± SD values are shown for *n* = 5 mice

Next, we assessed whether the StrandLock design affects interactions with the FcRn receptor, a key determinant of IgG half-life. bsFc-D retained pH-dependent binding to FcRn in vitro, displaying kinetics and affinity comparable to a control scIgG of WT atezolizumab **(**Fig. 5A-B**)**. Consistent with these results, in vivo pharmacokinetic analysis in SCID mice showed that bsFc-D exhibited a systemic exposure profile closely matching that of a 1:1 mixture of the parental scIgGs (atezolizumab and denintuzumab) **(**Fig. 5C**)**, indicating that β-strand D redistribution does not impair FcRn-mediated recycling or overall serum persistence.

### bsFc-D exhibits a favorable developability profile

**Fig. 6:**
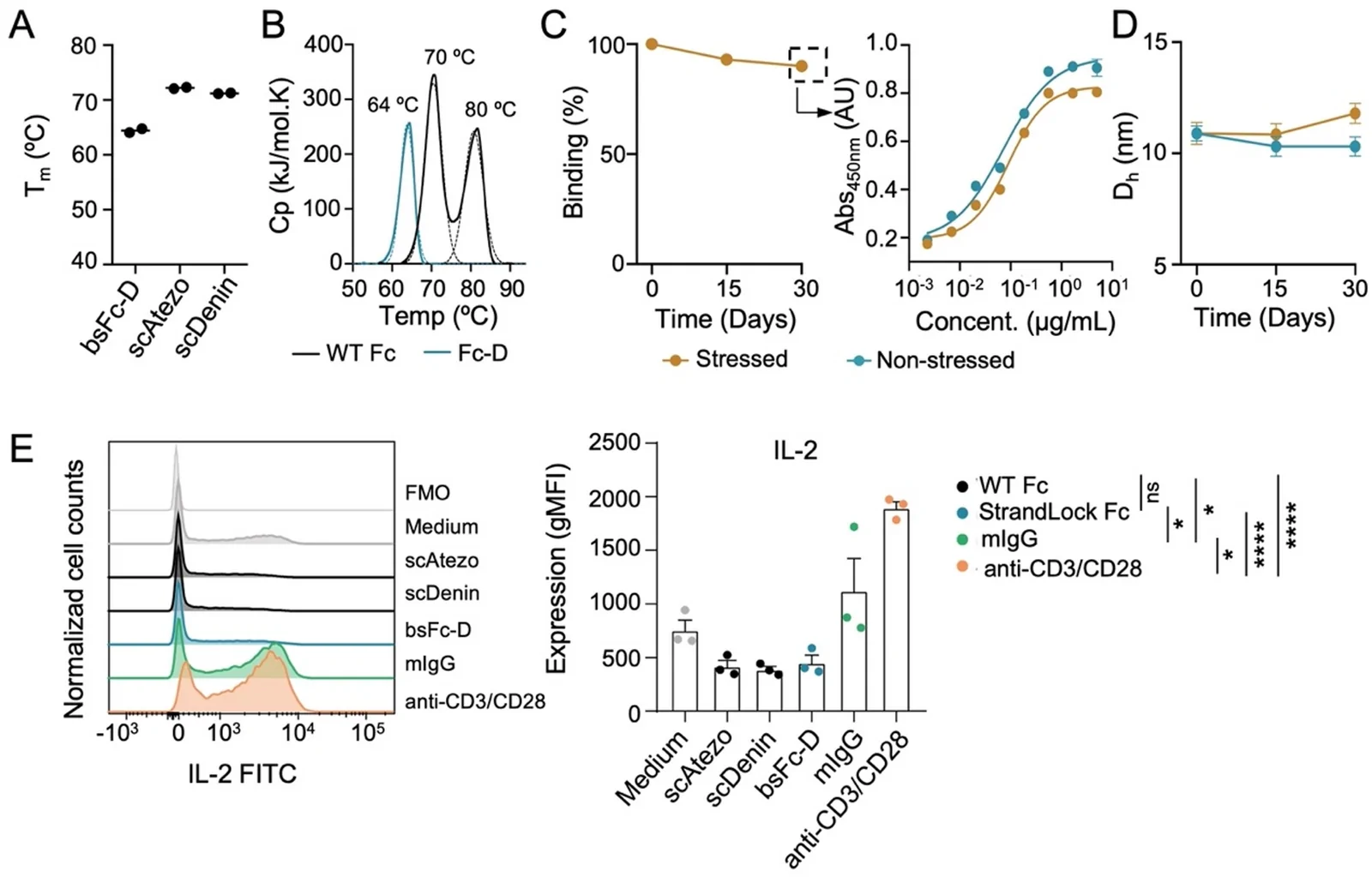
Biophysical and immunogenic characterization of bsFc-D. (**A**) T_m_ values of bsFc-D and parental monospecific scIgGs (atezolizumab and denintuzumab, WT Fc). (**B**) DSC thermograms of Fc fragments from bsFc-D (cyan) and atezolizumab (WT Fc, black) obtained by papain digestion. (**C**) Long-term stability of bsFc-D under stress conditions (10 mg/mL, 40 °C), evaluated by biweekly dual-antigen ELISA. Representative binding curves at week 4 compare stressed (orange) and non-stressed (cyan) samples. Data represent mean ± SD of two technical replicates. (**D**) Biweekly measurements of hydrodynamic diameter (D_h_) under the same stress conditions. Error bars indicate polydispersity of each measurement. (**E**) IL-2 production by human CD4^+^ T cells following incubation with parental WT antibodies or bsFc-D, measured by flow cytometry. Mouse IgG (mIgG) and anti-CD3/CD28 stimulation served as positive controls. Representative histograms of IL-2 quantification from 1 donor are shown (left; *n* = 3 donors). Not significant (ns, *p* > 0.05), * (*p* ≤ 0.05), and **** (*p* ≤ 0.0001)

The biophysical stability and resistance to stress of bsFc-D were next evaluated. Differential scanning calorimetry (DSC) showed a modest decrease of approximately 6 °C in the melting temperature (T_m_) of bsFc-D compared with the parental scIgGs (Fig. 6A and Fig. S5B). Notably, whereas the WT Fc domain exhibited two distinct unfolding transitions, corresponding to CH2 and CH3 domains, the engineered Fc displayed a single transition (Fig. 6B), likely reflecting altered domain cooperativity and a more integrated folding pattern resulting from chain interconnection.

Despite this thermal shift, the bsFc-D exhibited remarkable resilience under stress conditions. When concentrated to 10 mg/mL and stored at 40 °C for one month, the molecule retained full antigen-binding capacity. Simultaneous binding to PD-L1 and CD19 remained stable throughout the time course (days 0, 15, and 30), with no loss of activity relative to unstressed controls (Fig. 6C). Consistently, dynamic light scattering (DLS) confirmed the absence of aggregation during the stress period (Fig. 6D and Fig. S5C), reinforcing the physicochemical integrity of the molecule.

To further evaluate chemical stress stability under manufacturing-relevant conditions, bsFc-D was exposed to low-pH and repeated freeze-thaw cycling. Incubation at pH 3.5 for 2 h resulted in a moderate increase in high-molecular-weight species, although SEC analysis showed that ~ 80% of the sample retained the original profile. In contrast, bsFc-D exhibited full resistance to freeze-thaw stress, with no detectable aggregation or fragmentation after ten cycles by SEC analysis (Fig. S5D).

Finally, to assess whether the StrandLock-engineered Fc interface altered the immunogenic profile of the antibody, we first performed an in silico CD4⁺ T cell epitope prediction analysis using NetMHCIIpan 4.0 across nine prevalent HLA-DR alleles, providing broad population coverage [29, 30]. As expected, sequence regions with intrinsic immunoreactivity, such as Y^391^KTTPPVLD^399^, showed allele-dependent variability in predicted binding affinity. This effect was most evident in Fc_strand⁺ for HLA-DRB1*08:01, where several peptides already classified as strong binders in the WT Fc exhibited further decreases in %Rank values. Two additional sequences (F^405^LYSKLTVD^413^and L^410^TVDKSRWQ^418^) showed modest allele-specific shifts consistent with the introduced charge-reversal mutations (e.g., D399K/K409D and R416E). Importantly, these effects remained localized and did not translate into a global increase in predicted immunogenicity, as the engineered Fc variants did not exhibit an increased predicted CD4⁺ T cell epitope burden relative to the WT Fc across the evaluated HLA-DR alleles (Fig. S6).

To experimentally validate these predictions, we next performed an in vitro comparative immunogenicity assessment (IVCIA) using human PBMCs to evaluate CD4⁺ T cell activation through IL-2 production. Consistent with previous reports demonstrating the utility of this assay for assessing biologic immunogenicity [31, 32], strong IL-2 responses were detected in the positive controls, including a murine antibody, which elicited activation due to unmatched-species sequence differences, and anti-CD3/CD28 stimulation, used as a positive control for direct T-cell activation. In contrast, neither antibodies containing the WT Fc nor the StrandLock-engineered Fc variants induced detectable IL-2 secretion above background levels (Fig. 6E and Fig. S7). Together, these results demonstrate that the Fc mutations introduced in the StrandLock strategy do not enhance T cell immunogenicity, supporting the immunological safety of the engineered Fc interface.

### The StrandLock strategy demonstrates potential for broad applicability as a bsAb platform

**Fig. 7:**
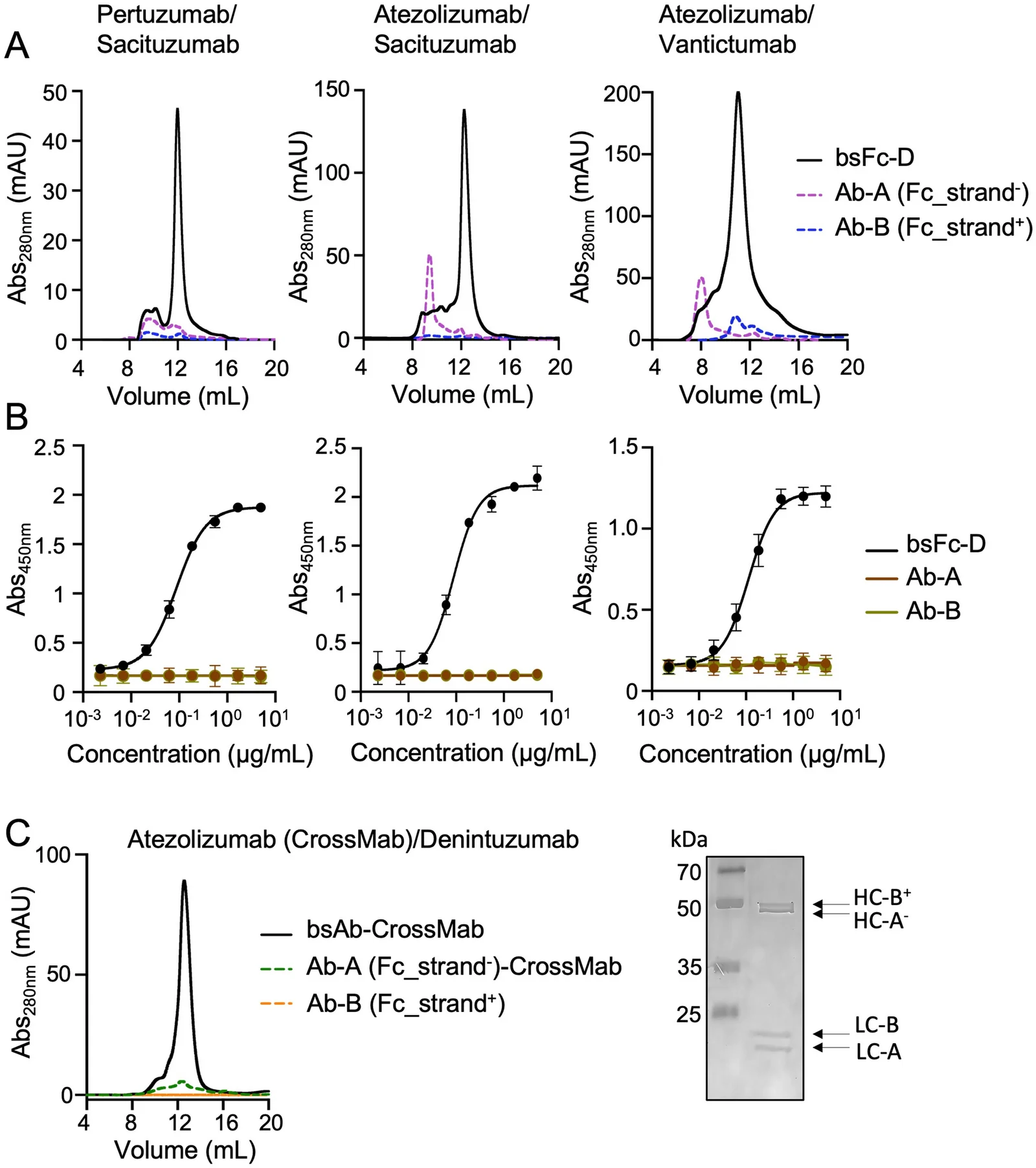
Versatility of the StrandLock strategy across different antibody specificities. (**A**) SEC profiles of the engineered bsFc-D with distinct antigen specificities (black), along with the corresponding monospecific scIgG constructs used in its generation: Ab-A^−^ (pertuzumab and atezolizumab; pink dashed) and Ab-B^+^ (sacituzumab and vantictumab; blue dashed). (**B**) Sandwich ELISA demonstrating the simultaneous binding capability of the engineered bsAbs, in contrast to the parental monospecific scIgG (Ab-A and Ab-B). (**C**) SEC profile and SDS-PAGE analysis of a bsAb generated by combining the StrandLock and CrossMab technologies (black). The corresponding SEC profiles of the monospecific antibodies are shown as dashed lines in green and orange

To evaluate the versatility of this strategy for bsAb generation, we extended its application beyond the initially validated denintuzumab-atezolizumab pair. This strategy was applied to three additional clinically relevant antibodies: sacituzumab (anti-trophoblast cell surface antigen-2 (TROP-2), pertuzumab (anti-human epidermal growth factor receptor 2 (HER2)), and vantictumab (anti-Frizzled-5 (FZD-5)), by engineering their Fc regions to incorporate the redistributed β-strand design. Various chain pairings were assessed for expression, proper assembly and bispecificity. In all tested combinations, the approach successfully yielded bsAbs capable of simultaneous antigen binding, with minimal formation of undesired homodimeric species (Fig. 7A-B).

To further assess the generalizability of the StrandLock design, we examined its compatibility with an independent LC expression system. In particular, we evaluated whether the redistributed β-strand architecture could be combined with the CrossMab format, a widely used strategy to enforce correct LC pairing [33]. When the individual antibody specificities were expressed separately, no detectable formation of undesired homodimeric species was observed, whereas co-transfection restored expression and yielded a discrete heterodimeric peak. Reducing SDS-PAGE confirmed the presence of both HC variants, distinguishable by their expected molecular-weight differences corresponding to the positive-strand and negative-strand Fc chains (Fig. 7C**)**.

Together, these findings illustrate the potential of the StrandLock technology to be applied across a diverse range of antibody specificities and to be integrated with complementary approaches to enable production of bsAbs.

## Discussion

BsAbs in IgG-like formats play a critical role in modern immunotherapy, as evidenced by multiple approved therapies and a robust clinical pipeline [34]. However, broader implementation and diversification of bsAb modalities remain constrained by key manufacturing challenges, most notably, the mispairing of HC and LC and undesired homodimer formation.

In this study, we present a structurally encoded Fc engineering platform that addresses these challenges through an unprecedented mechanism: interdependent folding via asymmetric β-strand redistribution. By redistributing a defined segment (residues N390–S400) between the CH3 domains of each HC, we disrupt the natural two-fold symmetry of the Fc dimer and impose a folding interdependency that establishes the structural framework for obligate heterodimerization. This approach marks a fundamental departure from traditional strategies, which typically rely on electrostatic steering or steric hindrance, to bias the CH3-CH3 interface toward heterodimer formation. In these platforms, including KiH and electrostatic steering, each chain retains, to varying degrees, the intrinsic capacity to assemble into homodimers, making mispaired species a possible outcome of expression. In contrast, the StrandLock design renders each HC structurally incomplete or unstable in isolation, thereby suppressing homodimer formation at the expression stage and ensuring that the only assembled species is the heterodimer. This mechanistic shift, from modulating interface preferences to enforcing chain interdependence, provides a robust, intrinsically encoded solution for bsAb assembly.

Building on this framework, we introduced a rational set of electrostatic mutations to further suppress homodimer formation and increase assembly fidelity. These modifications enabled the recovery of around 8 mg/L of bsAb, with purities approaching 99%, and allowed efficient recovery using a single affinity-based purification, comparable to that typically employed for conventional mAbs, without requiring additional steps to remove monospecific species. Notably, the resulting bsAb preserved the functional characteristics of native IgGs, including Fc receptor binding, immune effector functions, and pharmacokinetics. Furthermore, high-resolution x-ray crystallography confirms that despite the engineered asymmetry, the CH3 domain maintains native folding and packing.

While our strategy enhances control over heterodimer assembly and facilitates production, the introduction of non-native residues, may still represent a potential immunogenicity liability. To address this, we combined computational and experimental approaches to evaluate the immunogenic profile of the engineered Fc interface. In silico MHC class II binding predictions identified only localized and allele-dependent changes in peptide presentation, without increasing the overall predicted CD4⁺ T-cell epitope burden relative to the WT Fc. In parallel, preliminary IVCIA assays using human PBMCs showed no increase in IL-2 production upon exposure to bsFc-D compared with the parental WT antibodies, whereas the positive controls produced the expected activation response. Together, these results suggest that StrandLock engineering does not substantially enhance T-cell immunogenic potential despite the presence of engineered junctions, linker sequences, and charged interface mutations. Nevertheless, these studies represent an initial assessment, and additional long-term in vivo investigations will be required to comprehensively establish the immunological safety of this platform for therapeutic applications.

A key conceptual advance of this platform is the creation of an engineerable, asymmetric CH3-CH3 interface, a significant innovation compared to the symmetric homodimer interfaces used in current technologies. This asymmetry expands the design space for Fc engineering and creates new opportunities for computational approaches, including multistate design algorithms, AI-guided optimization of interface energetics, and deep learning-based sequence optimization tools that could systematically identify minimal mutational sets to maximize heterodimer yield while preserving favorable biophysical properties. This is particularly relevant given the modest reduction in thermal stability observed in our constructs (ΔT_m_ ~ 6 °C) and the moderate sensitivity to low-pH exposure, a stressor encountered during viral clearance. Nonetheless, the engineered bsAbs demonstrated remarkable resistance to aggregation under accelerated conditions, with no loss of binding capacity observed during the tested period.

Importantly, we demonstrate that the StrandLock technology is modular, with the potential for broad applicability. When incorporated into denintuzumab, atezolizumab, rituximab, sacituzumab, pertuzumab and vantictumab, the approach yielded well-folded functional molecules across a diverse set of target pairs. Although our current implementation uses a human IgG1 backbone, the strategy is conceptually compatible with other IgG isotypes (e.g., IgG2 and IgG4), which provide distinct effector functions and pharmacokinetic properties [35]. In addition, we showed that StrandLock can be combined with the CrossMab architecture [33] to control LC pairing, extending its applicability beyond the scIgG format and enabling its integration into conventional IgG designs with independently expressed LCs. Beyond this demonstrated case, the asymmetric Fc interface created by StrandLock should also be compatible with other strategies such as common LC technologies [21], offering opportunities for future expansion of the platform.

In summary, the StrandLock platform provides a mechanistically novel and robust solution to one of the central challenges in bsAb production, i.e., the generation of monospecific species. Its unique interdependent chain heterodimerization mechanism combined with structural integrity, functional preservation, and modularity, positions it as a powerful addition to the therapeutic antibody engineering toolkit.

## Conclusions

The StrandLock strategy establishes a structure-guided approach for enforcing obligate HC heterodimerization in bsAbs through asymmetric β-strand redistribution at the CH3 interface. By creating an interdependent folding architecture, this design intrinsically suppresses homodimer formation and enables high-fidelity assembly of bispecific molecules while maintaining compatibility with streamlined, single-step purification workflows. Structural, biochemical, and in vivo analyses demonstrate preservation of native Fc architecture, effector functions, and pharmacokinetic behavior, supporting the functional integrity of the engineered molecules. The successful implementation of StrandLock across multiple therapeutic antibody pairs highlights its modularity and adaptability within diverse bsAb contexts. Collectively, these findings support the StrandLock platform as a versatile framework for the efficient production of bsAbs.

## Materials and methods

### Antibody expression and purification

The genes encoding the LC and HC of the WT mAbs, the scIgG constructs(both with WT Fc domains and with engineered Fc variants), the IgG-based StrandLock constructs incorporating the CrossMab architecture, and the Fc-engineered heterodimerization formats used for comparison (KiH, Zymeworks, and electrostatic steering), were cloned into the pcDNA3.4 expression vector. Recombinant antibody production was carried out by transient transfection of human embryonic kidney (HEK)-293 F cells (Thermo Fisher Scientific). Cells were cultured in FreeStyle™ 293 Expression Medium at a starting density of 0.8 × 10⁶ cells/mL. For each 200 mL culture, 25 µg of each plasmid encoding the heavy and light chains was co-transfected using PEIpro^®^ (Polyplus Transfection) at a PEI: DNA mass ratio of 3:1. Cultures were incubated for 7 days at 37 °C with 8% CO₂, 70% relative humidity, and agitation at 140 rpm in a humidified orbital shaker. Post-expression, cell cultures were harvested by centrifugation and filtered through a 0.22 μm membrane (EMD Millipore) to obtain clarified supernatants. Antibodies were typically purified by a two-step procedure comprising protein A affinity chromatography followed by size exclusion chromatography (SEC). Protein A purification was performed on a 5 mL HiTrap column (Cytiva) equilibrated in phosphate-buffered saline (PBS; 137 mM NaCl, 2.7 mM KCl, 10 mM phosphate, pH 7.4). Bound antibodies were eluted with 20 mM glycine-HCl, pH 2.2, and eluates were immediately neutralized with 1 M Tris-HCl, pH 9.0. For constructs carrying an N-terminal Strep-II (WSHPQFEK) tag (as indicated in the text), a three-step purification workflow was employed. Following protein A chromatography, eluates were subjected to a second affinity step using a 5 mL StrepTrap HP column (Cytiva), pre-equilibrated with Strep buffer (100 mM Tris-HCl, pH 8.0, 150 mM NaCl, 1 mM EDTA). Elution was carried out with the same buffer supplemented with 100 mM D-desthiobiotin. In all cases, final polishing was performed by SEC on a Superdex 200 Increase 10/300 GL column (Cytiva) equilibrated in PBS. Purified antibodies were aliquoted and stored at -80 °C until use.

### Expression and purification of recombinant human receptors

The genes encoding the ectodomains of human PD-L1 (UniprotKB Q9NZQ7) (residues 19–238), CD19 (UniprotKB P15391) (residues 20–291), TROP-2 (UniprotKB P09758) (residues 27–274), HER2 (UniprotKB P04626) (residues 23–652), Fc gamma RI (hFcγRI) (UniprotKB P12314) (residues 19–292), FZD-5 (UniprotKB Q13467) (residues 28–150) and human neonatal Fc receptor (hFcRn) (UniprotKB P55899) (residues 27–297) were synthesized by GeneArt (Thermo Fisher Scientific) and cloned into the pHLsec mammalian expression vector. This vector includes a C-terminal hexahistidine (6x His) tag to facilitate downstream purification. For PD-L1, CD19, TROP-2, HER2 and FZD-5, the ectodomain sequences were followed by a tobacco etch virus (TEV) protease cleavage site and fused to a monomeric variant of the Venus fluorescent protein [36] to enhance glycoprotein expression and solubility. TROP-2, HER2 and FZD-5 constructs additionally carried a C-terminal Strep-II tag, whereas the CD19 construct carried an N-terminal Strep-II tag for ELISA-based validation assays.

Recombinant proteins were transiently expressed in HEK-293 F suspension cells as previously described for antibody production but using 50 µg of filtered plasmid DNA per 200 mL culture. Clarified supernatants after centrifugation were loaded onto a HisTrap Ni-NTA affinity chromatography column (GE Healthcare) at a flow rate of 4 mL/min. The column was washed with buffer containing 20 mM Tris-HCl (pH 8.0), 150 mM NaCl, and 5 mM imidazole to remove non-specifically bound proteins. Target receptors were eluted using a linear gradient of imidazole up to 500 mM. Protein-containing fractions were pooled, concentrated as needed, and subjected to further purification by size-exclusion chromatography on a Superdex 200 Increase column (GE Healthcare) equilibrated with 20 mM Tris-HCl (pH 8.0) and 150 mM NaCl.

### Dual antigen-binding assay by sequential capture ELISA

The simultaneous binding capacity of the engineered bsFc-D was evaluated using a sandwich ELISA. High-binding 96-well plates (Thermo Scientific) were coated overnight at 4 °C with 50 µL/well of recombinant PD-L1 or HER2 (2 µg/mL) in phosphate-buffered saline (PBS, pH 7.4). The next day, wells were washed with PBS supplemented with 0.05% Tween-20 (PBST) and blocked for 1 h at room temperature (RT) with 200 µL/well of 3% (w/v) bovine serum albumin (BSA; PanReac AppliChem) in PBS. After washing with PBST, 50 µL/well of serially diluted antibody samples were incubated for 1 h at RT. Unbound antibody was removed by washing twice with PBST, and 50 µL/well of Strep-tagged detection antigens (CD19, TROP-2, or FZD-5; 5 µg/mL in sample dilution buffer) were subsequently added and incubated for 1 h at RT. After two additional washes with PBST, bound Strep-tagged antigen was detected using 50 µL/well of horseradish peroxidase (HRP)-conjugated anti-StrepTag II antibody (Sigma-Aldrich, 71591-M) at 1:4000 dilution in sample dilution buffer, after incubation for 1 h at RT. Following three final washes with PBST, signal was developed using TMB substrate (Thermo Fisher Scientific, 34021) according to the manufacturer’s instructions. The reaction was stopped with 100 µL/well of 3 M HCl, and absorbance was measured at 450 nm using a GloMax^®^ Discover Microplate Reader (Promega, GM3000).

### Differential scanning calorimetry

Thermal stability of the engineered bsFc-D, along with the parental scIgGs (denintuzumab and atezolizumab) containing WT Fc domains, was assessed using Differential Scanning Calorimetry (DSC) using a NanoDSC instrument (TA Instruments, New Castle, DE, USA). Samples were prepared at a concentration of 1 g/L in degassed phosphate-buffered saline (PBS, pH 7.4), which was also used as the reference buffer. Prior to sample analysis, instrument baselines were established by performing four consecutive scans with degassed PBS in both the sample and reference cells. All scans, including baseline and sample measurements, were conducted from 20 °C to 100 °C at a constant scan rate of 1 °C/min under a pressure of 3 atm. Data acquisition and analysis were performed using the NanoAnalyze software (TA Instruments). After subtraction of the buffer-buffer baseline, the differential heat flow was converted to molar heat capacity (Cp). A sigmoidal baseline was fitted to the pre- and post-transition regions of each thermogram, and thermal transitions were analyzed by Gaussian deconvolution using an iterative fitting algorithm. The melting temperature (T_m_) was defined as the temperature corresponding to the maximum of each fitted Gaussian peak.

### Accelerated thermal stability assay

Accelerated stability studies were performed by incubating the engineered bsFc-D at 10 mg/mL in PBS (pH 7.4) at 40 °C for up to 30 days. Stability was assessed at day 0, day 15, and day 30 using Dynamic Light Scattering (DLS) to monitor potential aggregation via changes in hydrodynamic diameter, and a sandwich ELISA (described above) to evaluate maintenance of functional bispecificity. For each time point, stressed samples were compared to non-stressed control aliquots from the same production batch, stored at -80 °C and thawed immediately prior to analysis.

### Stress assays (low-pH and freeze-thaw)

For low-pH stress assays, purified bsAbs were adjusted to 1 mg/mL, acidified to pH 3.5 with glycine-HCl, incubated for 2 h at room temperature, neutralized with 1 M Tris-HCl pH 9.0, and analyzed by SEC on a Superdex 200 Increase 10/300 GL column. The percentage of correctly assembled bsAb remaining after stress was quantified by measuring the area under the curve (AUC) of the main SEC peak eluting at approximately 12 mL relative to the AUC corresponding to higher-molecular-weight species.

For freeze-thaw stress assays, purified bsAbs at 1 mg/mL were subjected to ten consecutive freeze-thaw cycles by snap-freezing in liquid nitrogen and thawing at 25 °C in a water bath. Samples were analyzed by SEC on a Superdex 200 Increase 10/300 GL column after the final cycle.

### Crystallization and X-ray data collection

To obtain the engineered Fc-D domain for structural characterization, the purified bispecific antibody was digested with papain (enzyme: antibody ratio of 1:20, w/w) for 1 h at 37 °C. The digestion mixture was subsequently applied to a KappaSelect affinity column (Cytiva) to retain Fab fragments, while the Fc-containing flow-through was collected. This fraction was further purified by size-exclusion chromatography on a Superdex 200 Increase 10/300 GL column (Cytiva) equilibrated with 20 mM Tris-HCl, pH 9.0, and 150 mM NaCl. The resulting Fc domain was concentrated to 6 mg/mL and subjected to crystallization screening using the sitting-drop vapor diffusion method. Crystals suitable for X-ray diffraction were obtained in a condition comprising 0.2 M ammonium formate, pH 6.6, and 20% (w/v) polyethylene glycol (PEG) 3350. Prior to data collection, crystals were cryoprotected by immersion in reservoir solution supplemented with 25% (v/v) glycerol, and flash-cooled in liquid nitrogen. X-ray diffraction data were collected at the XALOC beamline in ALBA synchrotron (Barcelona (Spain)). Data was processed using XDS [37] in the P212121 space group. The 3D structure was solved by molecular replacement using Phaser [38] using the human Fc structure (PDB ID: 2WAH) as the search model. Iterative cycles of model building and refinement were carried out using Coot [39] and Phenix.refine [40], respectively. Final model statistics are summarized in Supplementary Table 1. All BSA values reported were calculated using EMBL-EBI PDBePISA. The crystal structure reported in this manuscript, has been deposited in the Protein Data Bank (www.rcsb.org) (PDB ID: 29ZV).

In the PDB, residues are numbered continuously for both Fc chains, whereas in the manuscript the original sequence numbering is preserved to facilitate comparison with the WT sequence. Residues that are duplicated as a result of β-strand transfer are indicated with a prime (′), while deleted residues appear as gaps in the numbering. For example, in chain Fc_Strand⁺, the transferred strand D is labeled N390′–D400′ in the manuscript, whereas in the PDB these residues correspond to positions 402–412, including the linker residue G401. In chain Fc_Strand⁻, manuscript residue N389 is followed by an unlabeled GGGS linker and then residue D401. In the PDB, the linker is numbered 390–393, resulting in D401 being numbered as residue 394.

### Biolayer interferometry

Binding affinity measurements were performed by BLI using an Octet RED96 instrument (Sartorius) at 25 °C. Experiments were conducted in PBS, pH 7.4 supplemented with 0.1% (w/v) bovine serum albumin (BSA) and 0.002% (v/v) Tween-20 (PBST-BSA). Ni-NTA biosensors were employed to capture His-tagged hFcγRI or hFcRn proteins until a target binding signal of approximately 0.8 nm was reached. For FcRn interactions, an additional set of measurements was performed in 10 mM sodium phosphate buffer, 150 mM sodium chloride, pH 5.6, supplemented with 0.1% (w/v) BSA and 0.002% (v/v) Tween-20, to mimic the acidic environment of endosomal compartments relevant to receptor binding and recycling. After equilibration in the respective running buffer, association kinetics were recorded by immersing biosensors into wells containing two-fold serial dilutions of antibody (ranging from 100 nM to 3.125 nM), followed by dissociation in buffer-only wells. Both association and dissociation phases were monitored for 180–200 s. Raw data were reference-subtracted and globally fit to a 1:1 Langmuir binding model using Octet Analysis Studio software. Kinetic parameters including association rate constants (*k*_*on*_), dissociation rate constants (*k*_*off*_), and equilibrium dissociation constants *(K*_*d*_) were derived from the global fit.

### Assessment of antibody binding to cells by flow cytometry

WT RTX IgG and its engineered Fc variant (RTX Fc-D) were expressed by co-transfection of separate HC and LC plasmids as these non-bispecific antibodies do not pose a risk of HC/LC mismatch, and were fluorescently labeled with Alexa Fluor 488 via maleimide chemistry. Briefly, antibodies were prepared at a concentration of 2 mg/mL in PBS and reduced with 2.5 mM tris(2-carboxyethyl)phosphine (TCEP) for 1 h at RT. Following reduction, a 10-fold molar excess of Alexa Fluor 488 C5 maleimide (Thermo Fisher Scientific) was added and incubated for 1 h at RT in the dark. Unreacted dye was removed using a PD-10 desalting column (Cytiva) equilibrated in PBS. For flow cytometry analysis, 1 × 10⁶ Daudi B cells per sample were harvested by centrifugation at 400 ×g for 5 min at 4 °C, washed, and resuspended in PBS. Cells were incubated with 10 µg/mL of each fluorescently labeled antibody for 30 min at 4 °C in the dark. After incubation, cells were washed twice with PBS supplemented with 1% (w/v) BSA to remove unbound antibody and fluorescence intensity was measured using a CytoFLEX flow cytometer (Beckman Coulter). Data acquisition and analysis were performed using the Floreada.io web-based flow cytometry analysis platform.

### Quantification of C1q deposition

C1q binding to antibody-opsonized cells was quantified by flow cytometry. Daudi cells (2.5 × 10⁵ per well) were washed and resuspended in complement-inactivated RPMI 1640 medium. Cells were transferred to 96-well V-bottom plates (Corning) and incubated with 10 µg/mL of antibody for 5 min at 37 °C. Subsequently, human C1q (Sigma-Aldrich) was added to a final concentration of 10 µg/mL, and samples were incubated for 1 h at 37 °C in a humidified incubator with 5% CO₂. Following incubation, cells were washed three times with ice-cold staining buffer (PBS supplemented with 1% (w/v) BSA) to remove unbound C1q. Surface-bound C1q was detected by staining with a FITC-conjugated polyclonal rabbit anti-human C1q antibody (Thermo Fisher Scientific), diluted in staining buffer, and incubated for 30 min at RT in the dark. After two additional washes with staining buffer, cells were resuspended in the same buffer, and the FITC signal of the secondary antibody was measured using a CytoFLEX (Beckman) instrument. Data was analyzed using Floreada.io.

### Complement-dependent cytotoxicity assay

CDC activity was evaluated using a luminescence-based cell viability assay. Antibodies were serially diluted in RPMI 1640 medium supplemented with 10% heat-inactivated fetal bovine serum and added in duplicate to 96-well flat-bottom plates containing 5,000 target cells per well. Human complement serum (Innovative Research) was added to a final concentration of 10% (v/v), and plates were incubated for 4 h at 37 °C in a humidified 5% CO₂ incubator.

After incubation, cell viability was determined using the CellTiter-Glo^®^ 2.0 Assay (Promega), following the manufacturer’s protocol. Luminescence was recorded in opaque black 96-well plates (Sigma-Aldrich) using a GloMax^®^ Discover microplate reader (Promega, GM3000). CDC was quantified as a decrease in luminescent signal relative to control wells containing cells with complement alone.

### Pharmacokinetic study

The in vivo pharmacokinetic profile of the engineered bsAb and parental scIgG mixture containing the WT Fc were assessed in 8-week-old CB-17/Icr-*Prkdc*^scid^/Rj female mice (Janvier Labs). Animals were housed in ventilated cages under controlled conditions (12-hour light/dark cycle, 21–23 °C, 40–55% humidity). All experimental procedures complied with EU regulations for animal experimentation and were approved by the Ethics Committee on Animal Research (CEEA) of the Research and Development Center CID-CSIC (protocol OH 1696/2024). Mice received a single intraperitoneal injection (5 mg/kg) of either the engineered bsAb or a WT IgG mixture consisted of an equimolar combination of atezolizumab and denintuzumab, prepared at 0.5 mg/mL in PBS. Blood samples (15–20 µL) were collected via tail tip puncture at specified time points post-injection (day 0, 1, 3, 6, 8, 10, and 15), with topical application of 2% lidocaine gel for analgesia following each collection. Terminal blood samples were obtained by cardiac puncture under 4% isoflurane anesthesia. Serum was isolated and circulating antibody concentrations were quantified by ELISA. Specifically, 96-well Nickel-coated plates (Thermo Scientific, 15442) were coated overnight at 4 °C with an equimolar mixture of recombinant PD-L1 and CD19 (2.5 µg/mL each in PBS). Plates were washed with PBST and blocked with 3% (w/v) BSA in PBS for 1 h at RT. After washing, serum samples diluted 1:100 in PBS were added and incubated for 1 h at RT. Bound human IgG was detected using horseradish peroxidase (HRP)-conjugated goat anti-human IgG (Thermo Scientific, 62-8420) at a 1:3000 dilution for 1 h at RT. Following three PBST washes, the assay was developed using a TMB substrate kit according to the manufacturer’s instructions, and absorbance was measured at 450 nm. Quantification was performed using standard curves generated from known concentrations of the respective antibodies.

### In vitro comparative immunogenicity assessment (IVCIA) assays

Human peripheral blood mononuclear cells (PBMCs) were isolated from buffy coats of blood donors by Ficoll-Paque Plus (Cytiva) density centrifugation (1200 xg for 10 min at RT) using SepMate-50 tubes (StemCell). For the IVCIA assay, human PBMCs were cultured at 2 × 10^6^ total cells/mL in CTS OpTmizer T Cell Expansion media (Thermo Fisher) supplemented with 5% CTS Immune Cell serum replacement (Thermo Fisher), 2 mM GlutaMAX (Thermo Fisher), and 1% penicillin-streptomycin (P/S) (Thermo Fisher). PBMCs were cultured in the presence of each tested antibodies at 40 µg/mL. For T cell activation positive control, T cells were cultured either with 40 µg/mL mouse IgG2a Isotype control (Bioxcell) or with plates coated with 5 µg/mL anti-CD3 (BD Biosciences) and 1 µg/mL soluble anti-CD28 antibodies (Invitrogen). After five days of culture, CD4^+^ T cell IL-2 production was assessed.

### IL-2 production assessed by flow cytometry

Cells were stimulated with the eBioscience cell stimulation cocktail together with protein transport inhibitors (Invitrogen) for 4 h under standard culture conditions. After that, cells were collected, washed with PBS and stained with LIVE/DEAD fixable blue kit for 30 min at 4 °C and protected from light. Then, cells were stained with fluorochrome-conjugated antibodies targeting membrane markers for 30 min at 4 °C and protected from light. All samples were blocked before antibody staining with a TruStain FcX blocker (BioLegend) (1:50 in staining buffer) for 10 min at RT. Then, cells were fixed and permeabilized with intracellular fixation and permeabilization buffer set (Invitrogen) following the manufacturer’s protocol. Cells were washed and stained with fluorochrome-conjugated antibodies against intracellular markers in permeabilization buffer for 30 min at RT. Finally, cells were washed once in staining buffer and acquired on a FACSymphony flow cytometer (BD Biosciences). All centrifugation steps were performed at 450 xg for 5 min at 4 °C. The results were analyzed using FlowJo version 10 (BD Biosciences).

## Supplementary Information

Below is the link to the electronic supplementary material.

Supplementary Material 1

## Data Availability

All data are available in the main text or the supplementary materials. Additional materials are available to the scientific community upon reasonable request to the corresponding authors and the execution of a materials transfer agreement. The crystal structure has been deposited in the Protein Data Bank (www. rcsb.org) (PDB ID: 29ZV).
