# Supplementary material for: Engineering Fc heterodimerization via asymmetric β-strand reconfiguration for bispecific antibody assembly
Source: J Biol Eng. 2026 Jun 18;20:132. doi: 10.1186/s13036-026-00717-x (PMC13520406; doi:10.1186/s13036-026-00717-x)
Supplement: Supplementary file 1 [file 13036_2026_717_MOESM1_ESM.docx]

**Supplementary Materials**

**Engineering Fc heterodimerization via asymmetric β-strand reconfiguration for bispecific antibody assembly**

Arkaitz Cano^1, #^, Izaskun Morillo^2, #^, Ainhoa Goenaga^3^, María Elena Laugieri^4^, June Ereño-Orbea^4,5^, Ander de Blas^4^, Eunate Valdaliso-Díez^4^, Beatriz Apellaniz^1,6^, and Edurne Rujas^2,3,5,6^

^1^Department of Physiology, Faculty of Pharmacy, University of the Basque Country (UPV/EHU), 01006 Vitoria-Gasteiz, Spain.

^2^Instituto Biofisika (UPV/EHU, CSIC), University of the Basque Country, 48940, Leioa, Spain.

^3^Department of Pharmacy and Food Sciences, Faculty of Pharmacy, University of the Basque Country (UPV/EHU), 01006, Vitoria-Gasteiz, Spain.

^4^Cancer Glycoimmunology lab, Center for Cooperative Research in Biosciences (CIC bioGUNE), Basque Research and Technology Alliance (BRTA), 48160 Derio, Spain.

^5^Ikerbasque, Basque Foundation for Science, 48013 Bilbao, Spain.

^6^Bioaraba Health Research Institute, Microbiology, Infectious Disease, Antimicrobial Agents, and Gene Therapy Group, 01009, Vitoria-Gasteiz, Spain.

^#^These authors contributed equally

*Corresponding author. E-mail: [edurne.rujas@ehu.eus](mailto:edurne.rujas@ehu.eus)

**Fig. S1. Structural organization of the WT Fc. A)** Cartoon representation of the Fc structure (PDB ID: 3AVE) shown in light blue, with loop regions colored in gold. A schematic of β-strands (A–G) is included, displaying the corresponding amino acids. The inset shows a close-up view of opposing β-strand D within the CH3 domains. Side chains of residues N390 and S400, located at the C- and N-termini of the antiparallel β-strand D from adjacent monomers, respectively, are shown as sticks. The dashed black line indicates the distance between these residues, highlighting their close spatial proximity and the site of chain cleavage enabling β-strand D transfer between monomers. **B)** Cartoon representation of the Fc structure and schematic of the β-strands (A-G) for the StrandLock Fc. The interconnection between the two Fc chains (Fc_strand^-^ and Fc_strand^+^) is shown. Fc_strand^-^ (gray) and Fc_strand^+^ (dark red) are depicted with β-strands in bold. Transferred amino acids between chains are highlighted with a dotted box and indicated with a prime (′), while engineered linkers are shown in green. The amino acid sequence of A-G strands is displayed.

**Fig. S2. Assembly and purification of engineered antibody variants used for bsAb generation. A)** Overlapping SEC profiles of monospecific Ab-A^-^ mutants. The black arrow indicates the peak corresponding to correctly assembled IgGs. **B)** Protein A affinity chromatogram of engineered Ab-A^-^ (I253A mutant) showing no protein recovery after elution, confirming loss of protein A binding (yellow box). **C)** Schematic overview of the two-step purification strategy for bsAbs generated by co-transfection of Ab-A^-^ (I253A, His-tagged) and Ab-B^+^ (with or without additional mutations). The workflow involves protein A affinity purification followed by Ni-NTA chromatography. Representative examples include co-transfection of Ab-A^-^ containing no mutations (No mut., top) with Ab-B^+^ containing DK_KD_PK mutations (D399K_K409D_P396′K, bottom). **D)** Expression yields of bsAb formation after co-transfection of Ab-A^-^ (I253A mutant) with Ab-B⁺ variants carrying single or combined lysine substitutions. **E)** Protein A affinity chromatograms of individually expressed Ab-B^+^ mutants, demonstrating suppression of homodimer formation, as indicated by the absence of protein elution peaks (yellow boxes) in the last three variants.

**Fig. S3. Formation of monospecific homodimeric species in conventional Fc-engineered heterodimerization platforms.** Protein A affinity chromatograms and SEC profiles of the monospecific antibody species used for bsAb generation with the KiH **(A)**, electrostatic steering **(B)**, and Zymeworks **(C)** platforms. Yellow boxes highlight the peaks corresponding to homodimeric species.

**Fig S4**. **Overall structure and β-strand D organization of the Fc-D domain**. **A)** Cartoon representation of the Fc-D structure, with Fc_Strand^+^ shown in red and Fc_Strand^-^ in grey, highlighting the two N-linked glycans at position N297. **B)** Superposition of the crystal structures of Fc-D (red and grey) and WT Fc (PDB ID: 3AVE, black). **C)** Close-up view of β-strand D, with the 2FoFc electron density map shown as a blue mesh contoured at 1.0 σ.

**Fig. S5. Biophysical stability of bsFc-D. A)** Protein A affinity chromatograms of engineered monospecific RTX_strand⁺ and RTX_strand⁻ with minimal recovery (arrow), and SEC profile of properly folded RTX Fc-D. **B)** DSC profiles of monospecific scAtezolizumab and scDenintuzumab and the bsFc-D. Scans were recorded from 20 °C to 100 °C. T_m_ values of the main transitions are shown. Solid lines represent raw data; dashed lines correspond to fitted curves. **C)** DLS size distribution profiles of bsFc-D under stressed (40 °C, orange) and non-stressed conditions (cyan), measured at the beginning of the assay, after 2 weeks, and after 4 weeks. **D)** SEC profiles of bsFc-D after 2 h incubation at low pH 3.5 (left) and 10 freeze-thaw cycles.

**Fig. S6. In silico assessment of potential CD4⁺ T-cell epitopes in engineered Fc variants.** Predicted MHC class II binding profiles were generated using NetMHCIIpan-4.0 across nine prevalent HLA-DR alleles (HLA-DRB1*01:01, *03:01, *04:01, *07:01, *08:01, *09:01, *11:01, *13:01, and *15:01), collectively providing broad global population coverage. The Fc sequences were divided into overlapping 15-mer peptides, and each peptide was evaluated for its predicted binding affinity to the selected HLA-DR molecules. Binding predictions are reported as %Rank values, where lower values indicate a higher probability of peptide presentation. Dashed lines indicate the established NetMHCIIpan thresholds used to classify strong binders (%Rank < 2) and weak binders (%Rank < 10).

**Fig. S7.** Flow cytometry gating strategy to specifically analyse CD4^+^ T cell IL-2 production differentiating from the rest of PBMCs.

**Supporting Tables**

**Table S1. Crystallographic data collection and refinement statistics.**

| **bsFc-D** | |
| --- | --- |
| PDB ID | 29ZV |
| **Data collection Statistics** |  |
| Wavelength (Å) | 0.619923 |
| Resolution (Å) | 74.267- 2.064 (2.100 - 2.064) |
| Space group | P 21 21 21 |
| Unit cell dimensions |  |
| a, b, c (Å) | 49.64, 75.31 148.55 |
| α, β, γ (°) | 90, 90, 90 |
| Multiplicity | 11.9 (7.3) |
| Completeness (%) | 99.4 (88.5) |
| Mean I/σI | 10.3 (0.9) |
| Wilson B-factor (Å2) | 44.19 |
| Rmerge | 0.129 (2.627) |
| Rmeas | 0.135 (2.812) |
| Rpim | 0.039 (0.970) |
| CC1/2 | 99.8 (35.8) |
| **Refinement Statistics** |  |
| Resolution (Å) | 36.5- 2.04 |
| No. reflections | 36403 (3437) |
| Rwork/Rfree | 0.2285 /0.2784 |
| No. atoms: | 3593 |
| Protein | 3393 |
| Ligand | 170 |
| Solvent | 90 |
| RMS (bonds) | 0,003 |
| RMS (angles) | 0.68 |
| Ramachandran statistics: |  |
| favoured (%) | 98,04 |
| allowed (%) | 1.47 |
| outliers (%) | 0.49 |
| Rotamer outliers (%) | 0 |
| Average B (Å2): | 58.37 |
| Protein | 57.57 |
| Ligand | 79.03 |
| Solvent | 48.96 |
